# Association of log odds of positive lymph nodes with survival in patients with small cell lung cancer: Results from the SEER database

**DOI:** 10.1016/j.clinsp.2024.100369

**Published:** 2024-05-01

**Authors:** Ting Gao, Yingxuan Chang, Hongmei Yue

**Affiliations:** a The First Clinical Medical College of Lanzhou University, Gansu, P.R. China; b Department of Respiratory and Critical Care Medicine, Xianyang Central Hospital, Shaanxi, P.R. China

**Keywords:** SCLC, Lymph Node, Survival, LODDS, SEER

## Abstract

**Objectives:**

The prognosis of patients with Small Cell Lung Cancer (SCLC) can be predicted by their Lymph Node (LN) status. The authors aimed to assess the correlations between SCLC survival and number of LN Ratio (LNR), positive LN (pLNs), and Logarithmic Odds of positive LN (LODDS).

**Methods:**

This cohort study retrospectively included 1,762 patients with SCLC from the SEER database 2004‒2015. The X-tile software was used to determine the cutoff values for pLNs, LNR, and LODDS. The correlations between pLNs, LNR, and LODDS with Overall Survival (OS) and Cancer-Specific Survival (CSS) were explored using Cox regression analysis. The study used the C-index to assess the predictive value of LNR, pLNs, and LODDS on survival.

**Results:**

Among these 1,762 patients, 121 (6.87%) were alive, 1,641 (93.13%) died, and 1,532 (86.95%) died of SCLC. In univariable COX analysis, LNR, pLNs, and LODDS all showed a correlation with CSS and OS (p < 0.05). In multivariable COX analysis, only patients with LODDS (> 0.3 vs. ≤ 0.3) were related to both worse OS (HR = 1.28, 95% CI 1.10‒1.50) and CSS (HR = 1.29, 95% CI 1.10‒1.51), but no correction was observed between LNR and pLNs and survival (p > 0.05). The C-indices for predicting OS for LODDS were 0.552 (95% CI 0.541‒0.563), for LNR 0.504 (95% CI 0.501‒0.507), and for pLNs 0.527 (95% CI 0.514‒0.540). Moreover, the association between LODDS and prognosis in SCLC patients was significant only in patients with LN stage N1 and N2, but not in stage N3.

**Conclusion:**

LODDS may be better than other LN assessment tools at predicting survival in SCLC patients.

## Highlights

• The prognosis of patients with SCLC can be predicted by their LN status.
• The authors aimed to assess the correlations between SCLC survival and LNR, pLNs, LODDS.
• LODDS may be better than other LN assessment tools at predicting survival in SCLC.

## Introduction

Lung Cancer (LC) has a high global diagnosis and mortality, with two million people newly diagnosed and 1.76 million dying from LC each year.^1^ Although Small Cell Lung Cancer (SCLC) accounts for only 10%‒15% of lung cancers, it is a very aggressive disease and possesses a very low survival rate.^1^^,^^2^ Radiation and chemotherapy are the primary treatments for most people with SCLC.^3^ Surgical intervention is also recognized as an effective therapy for patients with early-stage SCLC.^4^^,^^5^ SCLC is sensitive to initial radiation and chemotherapy, but most patients will die of relapsing disease.^6^ Prognostic assessment is critical to the management of SCLC.

One of the bases for prognostic evaluation and therapeutic guidance in lung cancer is the American Joint Committee on Cancer (AJCC) staging system.^7^^,^^8^ However, N-staging was not updated with the AJCC system (8^th^ edition). N-staging is categorized by the anatomical location of positive Lymph Nodes (LNs), not the number of LNs.^9^ For lung cancer LN metastasis (N1, N2, N3), N1 refers to intrapulmonary LN metastasis (ipsilateral parabronchial or ipsilateral hilar and intrapulmonary LNs), while N2 and N3 refer to extrapulmonary LN metastasis (N2, ipsilateral mediastinal or sublung LNs; N3 contralateral mediastinal, contralateral hilar LNs, and ipsilateral or contralateral obliquus or supraclavicular LNs).^8^ The current N-staging has a poor ability to discriminate between patients for survival, and the number of positive LNs (pLNs) may be useful in the assessment of LN metastatic burden.^10^^,^^11^ In addition, several pieces of evidence suggested that adequate lymph node testing is more conducive to the prognostic assessment.^12^^,^^13^ Therefore, previous studies have constructed some indicators to evaluate the LN status of lung cancer, such as positive LN Ratio (LNR), pLNs, and the Log Odds of positive LNs (LODDS).^14^, ^15^, ^16^, ^17^ LNR has been used for risk stratification of lung cancer patients with prognostic value.^15^^,^^18^ However, pLNs and LNR do not allow risk stratification of patients without positive LN metastases. The LODDS uses formula transformation to stratify survival differences between patients by pathologic lymph node data, even in the absence of positive LNs. In patients with Non-SCLC (NSCLC), LODDS had better prognostic value than pLNs and LNR.^16^ Nevertheless, the relationships between LNR, pLNs, and LODDS and survival in patients with SCLC have been less reported.

Herein, this study was designed to evaluate the correlations between LNR, pLNs, and LODDS and survival in patients with SCLC and to analyze the effects of LNR, pLNs, and LODDS in predicting survival.

## Methods

The retrospective cohort study followed the STROBE Statement.

### Study design and patients

Patients were selected from the 2004‒2015 Surveillance, Epidemiology, and End Results (SEER) database (total of eighteen registries, Nov 2019 Sub [2000‒2017], released 2020). The National Cancer Institute's SEER program pulls information on the incidence of cancer and survival from eighteen registries across the United States. In the current retrospective cohort study, patients with a diagnosis of primary SCLC and an age at diagnosis of ≥18 years were included. SCLC patients were identified using the International Classification of Disease for Oncology, Third Edition (ICD-O-3) site codes (C34.0‒C34.9) and histological classification codes (8002/3, 8041‒8045/3). The following criteria were used to exclude patients: (1) With a diagnosis of SCLC based on autopsy or death certificate reports; (2) Two or more primary cancers; (3) LNs not examined; (4) Number of positive LNs unknown; and (5) Incomplete clinicopathological and survival information. The institutional ethical committee of Xianyang Central Hospital approved the study (nº 2023-IRB-70).

### Outcomes

The main outcomes were Overall Survival (OS) and Cancer-Specific Survival (CSS). SEER death information is captured by comparison with the National Death Index (NDI).^19^ The OS interval is the time between diagnosis and death due to any cause, and CSS interval is the time between diagnosis and death due to SCLC. Patients were followed from diagnosis until death, loss to follow-up, or administrative follow-up cessation (December 31, 2019).

### Variables and definition

The pLNs and the number of examined LNs are recorded directly in the SEER database. The calculation of LNR was: LNR = pLNs / the number of LNs examined. The calculation of LODDS: LODDS = log [(pLNs + 0.05) / (the number of LNs examined - pLNs + 0.05)]. Cutoff values for classifying LNR, pLNs, and LODDS were determined using X-Tile (v3.6.1).^20^ The pLNs, LNR, and LODDS were categorized as follows: pLNs (1 and > 1), LNR (≤ 0.05 and > 0.05), and LODDS (≤ 0.3 and > 0.3).

Other variables included patients’ age, race, gender, marital status, AJCC (8^th^ edition) TNM staging, laterality, grade of tumor, tumor size, primary location, surgery type, radiation, chemotherapy, and follow-up time. Laterality was classified as left, right, and others. Primary location was classified as upper lobe, middle lobe, lower lobe, overlapping lung lesion, main bronchus, and lung-NOS (Not Otherwise Specified). Surgery type was categorized as lobectomy, local tumor destruction, pneumonectomy, sublobectomy, no surgery, and surgery-NOS.

### Statistical analysis

The Wilcoxon rank-sum test was used for different analyses of continuous data, with medians and quartiles (Q1, Q3). Categorical data were reported as frequencies and percentages [n (%)]. Chi-Square or Fisher's exact tests were used for various analyses. Confounders related to OS and CSS were screened by the univariable Cox proportional hazards model (Supplementary Table 1).

Kaplan-Meier curve was used to analyze the survival of different LODDS and AJCC stage patients, and the comparison of differences was performed by log-rank test. The correlations of pLNs, LNR, and LODDS with survival were evaluated by a multivariable Cox proportional hazards model with Hazard Ratio (HR) and 95% Confidence Interval (95% CI) reported. The associations between pLNs, LNR, and LODDS and OS and CSS were further stratified by AJCC N staging. Furthermore, LNR, pLNs, and LODDS were evaluated for their ability to predict survival., and the predictive effect was assessed utilizing the Concordance-index (C-index) or the Area Under the Curve (AUC). The C-index estimates the probability that the predicted results are consistent with the actual observed results and is used to evaluate the predictive power of the model, where a C-index of 0.5 indicates that the model is not predictive and a C-index of 1 indicates that the model predictions are in perfect agreement with the actual results. Information on patients with SCLC from the SEER database was collected using SEER*STAT (v8.4.0). R software (v4.2.1) was used for statistical analyses, with p-values below 0.05 (two-tailed) considered to be statistically significant.

## Results

### Patient characteristics

**Figure 1:**
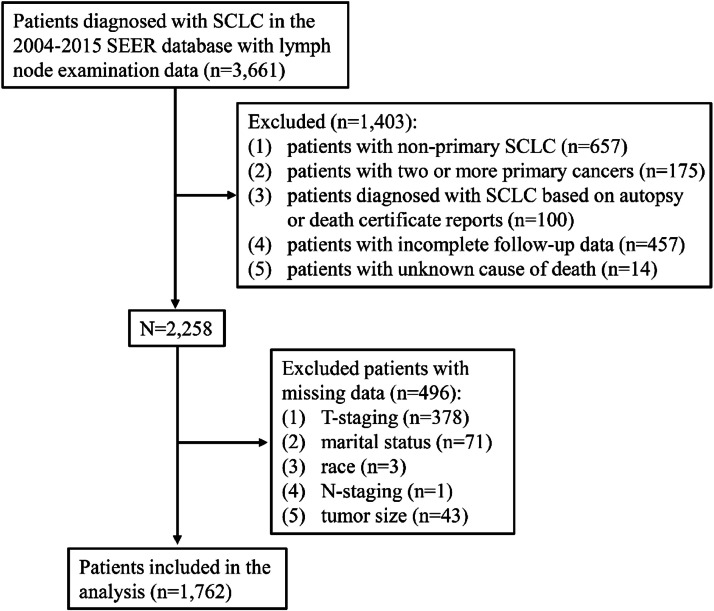
Flow chart for screening of study patients. SEER, the Surveillance, Epidemiology, and End Results database; SCLC, Small Cell Lung Cancer.

In total, 1,762 patients diagnosed with SCLC were included (Fig. 1). The characteristics of the patients with different AJCC N-stages are presented in Table 1. Among these patients, 897 (50.9%) were aged < 65-years, 908 (51.53%) were female, and 1,072 (60.84%) were in the right laterality. The most common primary tumor site was the upper lobe of the lung (48.07%), followed by the lower lobe of the lung (17.59%). For the treatment, 793 (45.01%) patients received radiotherapy, 1,405 (79.74%) patients received chemotherapy, and 1,506 (85.47%) patients received no surgery. The median length of follow-up was 11.00 (4.00, 20.00) months. Finally, 121 (6.87%) patients were alive, 1,641 (93.13%) patients died, and 1,532 (86.95%) patients died of SCLC. All patients had positive LNs, 1,174 (66.63%) had a positive LN count of 1 and 588 (33.37%) patients had a positive LN count > 1. There were 1,748 (99.21%) patients with LNR > 0.05 and 1,420 (80.59%) patients with LODDS > 0.3.

**Table 1:** Characteristics of SCLC patients with different AJCC N stages.

Variables	Total (n = 1762)	N1 stage (n = 184)	N2 stage (n = 999)	N3 stage (n = 579)	p
Age, years, n (%)					0.002
< 65	897 (50.91)	108 (58.70)	526 (52.65)	263 (45.42)	
≥ 65	865 (49.09)	76 (41.30)	473 (47.35)	316 (54.58)	
Sex, n (%)					0.625
Female	908 (51.53)	101 (54.89)	512 (51.25)	295 (50.95)	
Male	854 (48.47)	83 (45.11)	487 (48.75)	284 (49.05)	
Race, n (%)					0.115
American Indian/Alaska Native	13 (0.74)	0 (0.00)	8 (0.80)	5 (0.86)	
Asian or Pacific Islander	75 (4.26)	4 (2.17)	38 (3.80)	33 (5.70)	
Black	154 (8.74)	12 (6.52)	90 (9.01)	52 (8.98)	
White	1520 (86.27)	168 (91.30)	863 (86.39)	489 (84.46)	
Marital status, n (%)					0.089
Married	979 (55.56)	116 (63.04)	559 (55.96)	304 (52.50)	
Others	568 (32.24)	51 (27.72)	325 (32.53)	192 (33.16)	
Single	215 (12.20)	17 (9.24)	115 (11.51)	83 (14.34)	
AJCC T, n (%)					<0.001
T1	328 (18.62)	61 (33.15)	210 (21.02)	57 (9.84)	
T2	450 (25.54)	63 (34.24)	275 (27.53)	112 (19.34)	
T3	83 (4.71)	9 (4.89)	46 (4.60)	28 (4.84)	
TX	901 (51.14)	51 (27.72)	468 (46.85)	382 (65.98)	
AJCC M, n (%)					<0.001
M0	913 (51.82)	143 (77.72)	542 (54.25)	228 (39.38)	
M1	807 (45.80)	36 (19.57)	436 (43.64)	335 (57.86)	
MX	42 (2.38)	5 (2.72)	21 (2.10)	16 (2.76)	
Laterality, n (%)					0.002
Left ‒ origin of primary	627 (35.58)	77 (41.85)	337 (33.73)	213 (36.79)	
Right ‒ origin of primary	1072 (60.84)	103 (55.98)	636 (63.66)	333 (57.51)	
Others	63 (3.58)	4 (2.17)	26 (2.60)	33 (5.70)	
Tumor grade, n (%)					<0.001
Grade I	4 (0.23)	0 (0.00)	4 (0.40)	0 (0.00)	
Grade II	14 (0.79)	5 (2.72)	7 (0.70)	2 (0.35)	
Grade III	193 (10.95)	38 (20.65)	115 (11.51)	40 (6.91)	
Grade IV	297 (16.86)	56 (30.43)	166 (16.62)	75 (12.95)	
Unknown	1254 (71.17)	85 (46.20)	707 (70.77)	462 (79.79)	
Tumor size, n (%)					<0.001
≤ 50 mm	1365 (77.47)	163 (88.59)	790 (79.08)	412 (71.16)	
> 50 mm	397 (22.53)	21 (11.41)	209 (20.92)	167 (28.84)	
Primary Site, n (%)					<0.001
Main bronchus	193 (10.95)	10 (5.43)	118 (11.81)	65 (11.23)	
Upper lobe, lung	847 (48.07)	92 (50.00)	500 (50.05)	255 (44.04)	
Middle lobe, lung	83 (4.71)	10 (5.43)	45 (4.50)	28 (4.84)	
Lower lobe, lung	310 (17.59)	51 (27.72)	169 (16.92)	90 (15.54)	
Overlapping lesion of lung	28 (1.59)	6 (3.26)	8 (0.80)	14 (2.42)	
Lung (NOS)	301 (17.08)	15 (8.15)	159 (15.92)	127 (21.93)	
Surgery type, n (%)					<0.001
Lobectomy	157 (8.91)	89 (48.37)	66 (6.61)	2 (0.35)	
Local tumor destruction	1 (0.06)	0 (0.00)	0 (0.00)	1 (0.17)	
No surgery	1506 (85.47)	64 (34.78)	876 (87.69)	566 (97.75)	
Pneumonectomy	15 (0.85)	9 (4.89)	6 (0.60)	0 (0.00)	
Sublobectomy	74 (4.20)	21 (11.41)	45 (4.50)	8 (1.38)	
Surgery (NOS)	9 (0.51)	1 (0.54)	6 (0.60)	2 (0.35)	
Radiation, n (%)					0.015
No	969 (54.99)	89 (48.37)	578 (57.86)	302 (52.16)	
Yes	793 (45.01)	95 (51.63)	421 (42.14)	277 (47.84)	
Chemotherapy, n (%)					0.150
No	357 (20.26)	44 (23.91)	187 (18.72)	126 (21.76)	
Yes	1405 (79.74)	140 (76.09)	812 (81.28)	453 (78.24)	
Follow-up time, M (Q_1_, Q_3_)	11.00 (4.00, 20.00)	16.50 (7.00, 32.00)	11.00 (5.00, 22.00)	8.00 (3.00, 14.00)	<0.001
pLNs, n (%)					<0.001
1	1174 (66.63)	111 (60.33)	619 (61.96)	444 (76.68)	
> 1	588 (33.37)	73 (39.67)	380 (38.04)	135 (23.32)	
LNR n (%)					<0.001
≤ 0.05	14 (0.79)	9 (4.89)	5 (0.50)	0 (0.00)	
> 0.05	1748 (99.21)	175 (95.11)	994 (99.50)	579 (100.00)	
LODDS, n (%)					<0.001
≤ 0.3	342 (19.41)	110 (59.78)	190 (19.02)	42 (7.25)	
> 0.3	1420 (80.59)	74 (40.22)	809 (80.98)	537 (92.75)	
OS, n (%)					<0.001
Yes	121 (6.87)	28 (15.22)	79 (7.91)	14 (2.42)	
No	1641 (93.13)	156 (84.78)	920 (92.09)	565 (97.58)	
CSS, n (%)					<0.001
Yes	230 (13.05)	37 (20.11)	149 (14.91)	44 (7.60)	
No	1532 (86.95)	147 (79.89)	850 (85.09)	535 (92.40)	

SCLC, Small Cell Lung Cancer; AJCC, American Joint Committee on Cancer; TX, T-staging is not judgmental; MX, M-staging is not judgmental; NOS, Not Otherwise Specified; pLNs, the number of positive Lymph Nodes; LNR, positive Lymph Node Ratio; LODDS, the Log Odds of Positive lymph nodes; OS, Overall Survival; CSS, Cancer-Specific Survival.

### Correlations of pLNs, LNR, and LODDS with survival in SCLC patients

**Figure 2:**
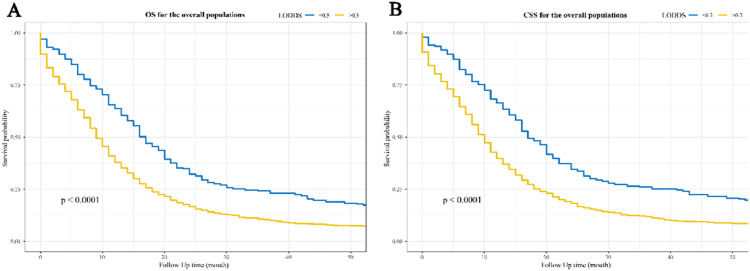
Kaplan-Meier survival curves for the impact of LODDS on OS and CSS in patients with SCLC. (A) OS for the overall populations; (B) CSS for the overall populations. LODDS, the Log Odds of positive lymph nodes; OS, Overall Survival; CSS, Cancer-Specific Survival; SCLC, Small Cell Lung Cancer.

**Figure 3:**
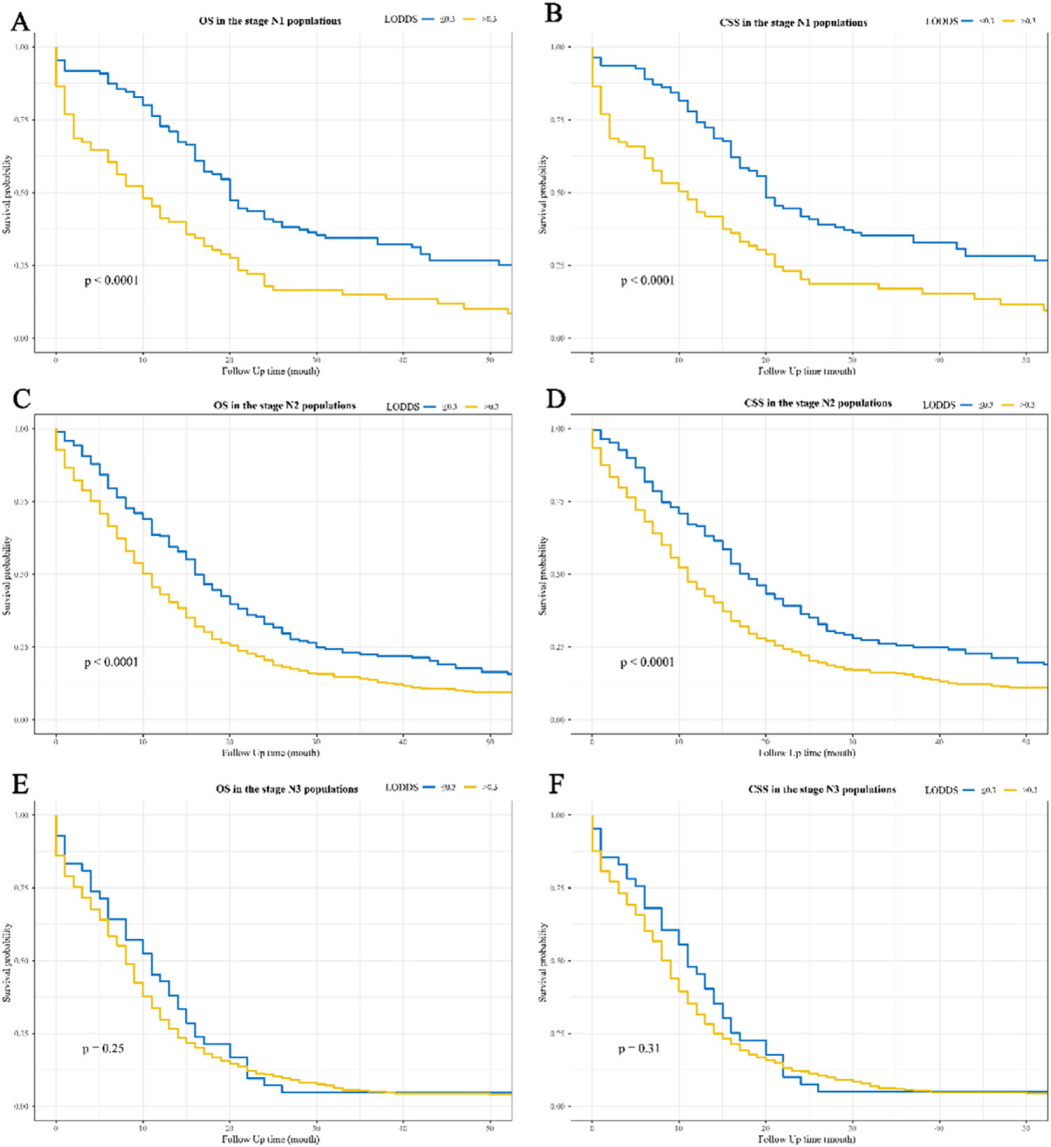
Kaplan-Meier survival curves for the impact of LODDS on OS and CSS in SCLC patients with different N stages. (A) OS in the stage N1 populations; (B) CSS in the stage N1 populations; (C) OS in the stage N2 populations; (D) CSS in the stage N2 populations; (E) OS in the stage N3 populations; (F) CSS in the stage N3 populations. LODDS, the Log Odds of positive lymph nodes; OS, Overall Survival; CSS, Cancer-Specific Survival; SCLC, Small Cell Lung Cancer.

Figure 2 and Figure 3 show the Kaplan-Meier curves for OS and CSS affected by LODDS. LODDS > 0.3 were linked to poorer OS and CSS compared to LODDS ≤ 0.3 in the overall (Fig. 2A‒2B), stage N1 (Fig. 3A‒3B), and stage N2 (Fig. 3C‒3D) populations, but not in the stage N3 populations (Fig. 3E‒3F).

Table 2 lists the correlations of pLNs, LNR, and LODDS with survival. Patients with LODDS > 0.3 (vs. ≤ 0.3) (HR = 1.69; 95% CI 1.49‒1.92) or LNR > 0.05 (vs*.* ≤ 0.05) (HR = 2.37; 95% CI 1.27‒4.42)) had worse OS in the univariable COX analysis, whereas patients with pLNs > 1 (vs. 1) (HR = 0.84; 95% CI 0.76‒0.93) had better OS. After adjusting for confounders, only patients with LODDS > 0.3 (HR = 1.28; 95% CI 1.10‒1.50) had poorer OS, but LNR (p = 0.207) and pLNs (p = 0.482) showed no correlation with OS. Similarly, LODDS > 0.3 (HR = 1.71; 95% CI 1.50‒1.95) or LNR > 0.05 (HR = 2.19; 95% CI 1.17‒4.07) was correlated with worse OS in the univariable COX analysis, whereas patients with pLNs > 1 (HR = 0.81; 95% CI 0.73‒0.90) had a better OS. On multivariable Cox analysis, only patients with LODDS > 0.3 had a worse OS (HR = 1.29; 95% CI 1.10‒1.51).

**Table 2:** The correlations of pLNs, LNR, and LODDS with OS and CSS in patients with SCLC.

		Univariable analysis		Multivariable analysis	
Outcomes	Variables	HR (95%CI)	p	HR (95%CI)	p
OS	LODDS				
	≤ 0.3	Ref		Ref	
	> 0.3	1.69 (1.49‒1.92)	<0.001	1.28 (1.10‒1.50)	0.001
	pLNs				
	1	Ref		Ref	
	> 1	0.84 (0.76‒0.93)	<0.001	0.96 (0.87‒1.07)	0.482
	LNR				
	≤ 0.05	Ref		Ref	
	> 0.05	2.37 (1.27‒4.42)	0.006	1.51 (0.80‒2.86)	0.207
CSS	LODDS				
	≤ 0.3	Ref		Ref	
	> 0.3	1.71 (1.50‒1.95)	<0.001	1.29 (1.10‒1.51)	0.002
	pLNs				
	1	Ref		Ref	
	> 1	0.81 (0.73‒0.90)	<0.001	0.93 (0.83‒1.04)	0.191
	LNR				
	≤ 0.05	Ref		Ref	
	> 0.05	2.19 (1.17‒4.07)	0.014	1.38 (0.73‒2.62)	0.322

pLNs, the number of positive Lymph Nodes; LNR, positive Lymph Node Ratio; LODDS, the Log Odds of positive lymph nodes; OS, Overall Survival; CSS, Cancer-Specific Survival; SCLC, Small Cell Lung Cancer; Ref, Reference; HR, Hazard Ratio; CI, Confidence Interval; Multivariable Cox regression analysis adjusted for age, sex, marital status (not adjusted when analyzing CSS), AJCC TNM, tumor size, laterality, surgery type, radiation, and chemotherapy.

The associations between LNR, pLNs, and LODDS and OS and CS were stratified according to N-staging (Table 3). Patients with LODDS > 0.3 had poorer OS in the stage N1 (HR = 1.60; 95% CI 1.01‒2.54)) and stage N2 (HR = 1.33; 95% CI 1.09‒1.61) populations, but not in the stage N3 (p = 0.601) populations. In the CSS analysis, only in the N2 stage population did patients with LODDS > 0.3 have worse CSS. (HR = 1.34; 95% CI 1.09‒1.65)), whereas there were no associations between LODDS and CSS in the N1 (p = 0.100) and N3 (p = 0.606) populations. Furthermore, no associations of pLNs and LNR with OS and CSS were found in the stages N1 (p > 0.05), N2 (p > 0.05), and N3 (p > 0.05) populations.

**Table 3:** Stratified analysis of the relationships of pLNs, LNR, and LODDS with OS and CS according to N-staging.

			Univariable analysis		Multivariable analysis	
Outcomes	N-staging	Variables	HR (95%CI)	P	HR (95%CI)	P
OS	N1	LODDS				
		≤ 0.3	Ref		Ref	
		> 0.3	2.02 (1.47-2.78)	<0.001	1.60 (1.01-2.54)	0.044
		pLNs				
		1	Ref		Ref	
		> 1	0.78 (0.56-1.08)	0.135	0.97 (0.67-1.41)	0.881
		LNR				
		≤ 0.05	Ref		Ref	
		> 0.05	1.26 (0.59-2.70)	0.545	1.09 (0.47-2.52)	0.836
	N2	LODDS				
		≤ 0.3	Ref		Ref	
		> 0.3	1.46 (1.23-1.72)	<0.001	1.33 (1.09-1.61)	0.005
		pLNs				
		1	Ref		Ref	
		> 1	0.92 (0.80-1.05)	0.212	0.99 (0.86-1.13)	0.834
		LNR				
		≤ 0.05	Ref		Ref	
		> 0.05	3.44 (1.11-10.68)	0.033	2.26 (0.72-7.10)	0.164
	N3	LODDS				
		≤ 0.3	Ref		Ref	
		> 0.3	1.20 (0.87-1.66)	0.261	1.09 (0.79-1.51)	0.601
		pLNs				
		1	Ref		Ref	
		> 1	0.89 (0.73-1.08)	0.224	0.90 (0.73-1.10)	0.312
		LNR				
		≤ 0.05	-		-	
		> 0.05	-	-	-	-
CSS	N1	LODDS				
		≤ 0.3	Ref		Ref	
		> 0.3	1.97 (1.42-2.73)	<0.001	1.48 (0.93-2.37)	0.100
		pLNs				
		1	Ref		Ref	
		> 1	0.80 (0.57-1.11)	0.184	1.03 (0.70-1.50)	0.888
		LNR				
		≤ 0.05	Ref		Ref	
		> 0.05	1.19 (0.56-2.55)	0.652	1.02 (0.44-2.36)	0.96
	N2	LODDS				
		≤ 0.3	Ref		Ref	
		> 0.3	1.50 (1.26-1.79)	<0.001	1.34 (1.09-1.65)	0.005
		pLNs				
		1	Ref		Ref	
		> 1	0.87 (0.76-1.00)	0.053	0.93 (0.80-1.07)	0.324
		LNR				
		≤ 0.05	Ref		Ref	
		> 0.05	3.13 (1.01-9.73)	0.049	1.99 (0.63-6.27)	0.240
	N3	LODDS				
		≤ 0.3	Ref		Ref	
		> 0.3	1.18 (0.85-1.65)	0.314	1.09 (0.78-1.53)	0.606
		pLNs				
		1	Ref		Ref	
		> 1	0.87 (0.71-1.06)	0.170	0.90 (0.73-1.11)	0.310
		LNR				
		≤ 0.05	-		-	
		> 0.05	-		-	-

pLNs, the number of positive Lymph Nodes; LNR, positive Lymph Node Ratio; LODDS, the Log Odds of positive lymph nodes; OS, Overall Survival; CSS, Cancer-Specific Survival; SCLC, Small Cell Lung Cancer; Ref, Reference; HR, Hazard Ratio; CI, Confidence Interval; Multivariable Cox regression analysis adjusted for age, sex, marital status (not adjusted when analyzing CSS), AJCC TNM, tumor size, laterality, surgery type, radiation, and chemotherapy.

### Prediction effect of pLNs, LNR, and LODDS on survival

**Figure 4:**
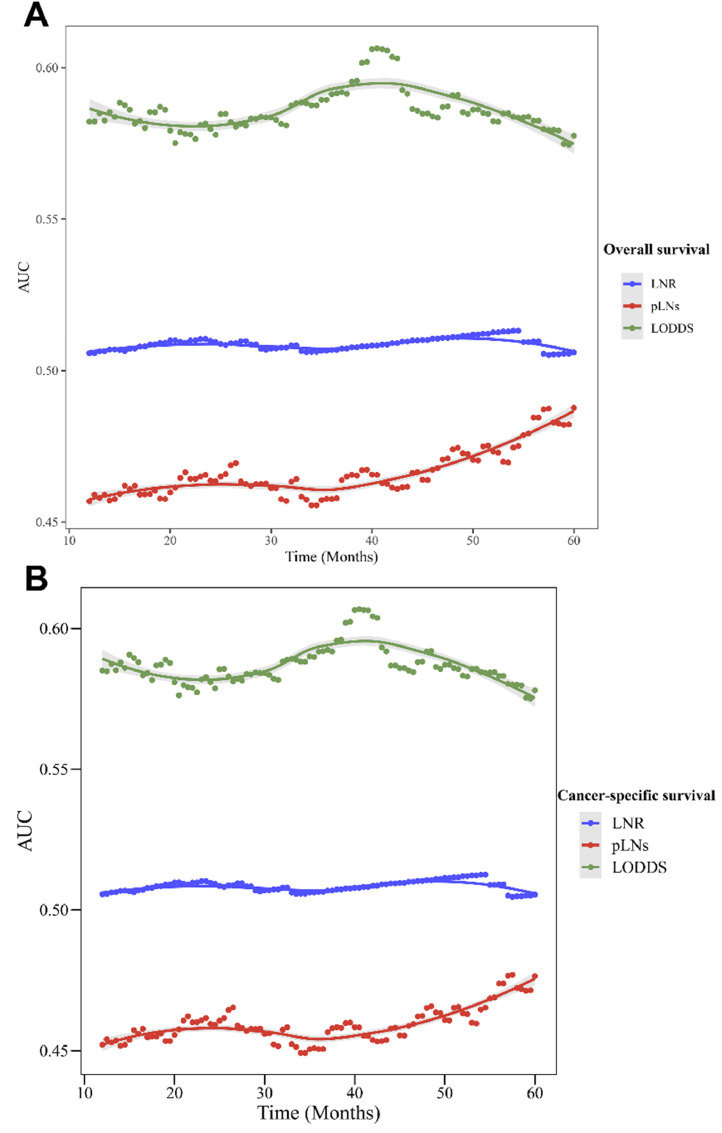
The changes in the Area Under the Curve (AUC) over time for OS and CSS predicted by pLNs, LNR, and LODDS. (A) Changes in AUC for predicting OS; (B) Changes in AUC for predicting CSS. OS, Overall Survival; CSS, Cancer-Specific Survival; pLNs, the number of positive Lymph Nodes; LNR, positive Lymph Node Ratio; LODDS, the Log Odds of positive lymph nodes.

Table 4 presents the prediction role of LNR, pLNs, and LODDS on OS and CSS for the overall and different N-stage populations. LODDS had a higher C index in the prediction of OS and CSS (OS: C-index = 0.552 [95% CI 0.541‒0.563]; CSS: C-index = 0.554 [95% CI 0.543‒0.565]) than pLNs and LNR in the overall populations (p < 0.05). The C-index of LODDS to predict OS in the N1, N2, and N3 populations was 0.601 (95% CI 0.560‒0.641), 0.540 (95% CI 0.525‒0.554), and 0.509 (95% CI 0.497‒0.522), respectively. The C-index of LODDS to predict CSS in the stage N1, stage N2, and stage N3 populations was 0.602 (95% CI 0.560‒0.643), 0.543 (95% CI 0.528‒0.557), and 0.511 (95% CI 0.498‒0.523), respectively. Figure 4 shows the changes in AUC over time for OS and CSS predicted by pLNs, LNR, and LODDS. The results demonstrated that LODDS predicted that the AUC of OS and CSS in SCLC patients would change over time more than pLNs and LNR.

**Table 4:** The prediction effect of pLNs, LNR, and LODDS on OS and CSS in patients with SCLC.

Outcomes	Populations	Variables	C-index (95%CI)	*P*
OS	Overall	LODDS	0.552 (0.541-0.563)	Ref
		pLNs	0.527 (0.514-0.540)	0.004
		LNR	0.504 (0.501-0.507)	<0.001
	N1 stage	LODDS	0.601 (0.560-0.641)	Ref
		pLNs	0.545 (0.502-0.588)	0.065
		LNR	0.505 (0.486-0.525)	<0.001
	N2 stage	LODDS	0.540 (0.525-0.554)	Ref
		pLNs	0.514 (0.496-0.532)	0.030
		LNR	0.504 (0.500-0.507)	<0.001
	N3 stage	LODDS	0.509 (0.497-0.522)	Ref
		pLNs	0.521 (0.500-0.541)	0.331
		LNR	-	
CSS	Overall	LODDS	0.554 (0.543-0.565)	Ref
		pLNs	0.531 (0.518-0.545)	0.008
		LNR	0.504 (0.501-0.507)	<0.001
	N1 stage	LODDS	0.602 (0.560-0.643)	Ref
		pLNs	0.543 (0.499-0.586)	0.057
		LNR	0.504 (0.484-0.525)	<0.001
	N2 stage	LODDS	0.543 (0.528-0.557)	Ref
		pLNs	0.520 (0.501-0.539)	0.063
		LNR	0.504 (0.500-0.507)	<0.001
	N3 stage	LODDS	0.511 (0.498-0.523)	Ref
		pLNs	0.523 (0.502-0.544)	0.341
		LNR	-	-

pLNs, the number of positive Lymph Nodes; LNR, positive Lymph Node Ratio; LODDS, the Log Odds of positive lymph nodes; OS, Overall Survival; CSS, Cancer-Specific Survival; SCLC, Small Cell Lung Cancer; Ref, Reference; CI, Confidence Interval.

## Discussion

LNs can be used to help predict the prognosis of SCLC patients. The relationship was analyzed between three evaluation indexes of lymph node status (LNR, pLNs, and LODDS) and the survival rate of SCLC patients. The results demonstrated that LNR, pLNs, and LODDS were correlated with OS and CSS in SCLC in univariable analyses, whereas only LODDS remained linked to OS and CSS in multivariable analyses. Higher values of LODDS were related to worse OS and CSS in SCLC. LODDS predicted survival in SCLC patients better than pLN and LNR. In addition, the relationship between LODDS and prognosis in SCLC patients was significant only in patients with LN stage N1 and N2, but not in stage N3.

N-staging is the most applied tool for evaluating the LN status.^21^ Nevertheless, N-staging in the AJCC staging system (including the latest 8^th^ edition) is according to anatomic location only, and there is no information on the number of LNs involved.^9^ The specific anatomical location and the number of involved LNs are important for the LC prognosis.^22^, ^23^, ^24^ Moreover, the weakness of anatomically based N-staging is the potential for subjective physician judgment in classifying LNs based on the boundaries of anatomical location.^25^^,^^26^ Indicators for assessing the positive LNs (e.g., pLNs, LNR, and LODDS) in lung cancer patients have been proposed.^15^, ^16^, ^17^ Yang et al. showed that higher LNR values were linked to reduced OS and CSS in early-stage SCLC patients with surgically resected.^15^ Deng et al. indicated that pLNs, LNR, and LODDS were factors that influence survival in NSCLC patients.^16^ The authors evaluated the relationships of pLNs, LNR, and LODDS with survival in SCLC and showed that after adjusting for confounders, only high values of LODDS were linked to poorer OS and CSS in SCLC patients. However, a recent study reported that LNR, pLNs, and LODDS were linked to survival in SCLC after surgery.^17^ The inconsistent results of this study and previous studies regarding the effects of pLNs and LNR on survival in SCLC patients may be influenced by the severity of the disease. Previous studies included patients who underwent surgery, and these were usually early-stage patients, whereas in the present study 85.47% of the patients had no surgery and just 5.90% of the patients had an AJCC stage of Ⅰ/Ⅱ. This may indicate that the LODDS is a more applicable indicator than pLNs and LNR for evaluating lymph node status in the general population.

Because N-staging in the AJCC staging system is according to anatomical location, the authors further analyzed the correlation of LODDS with survival in SCLC patients across different N-staging populations. The results demonstrated that high LODDS values were related to worse OS in the stage N1 and stage N2 populations, whereas there was no association between LODDS and OS in the stage N3 populations. Stage N3 in lung cancer represents the involvement of the contralateral hilar, contralateral mediastinal, or supraclavicular nodes. The correlation between LODDS and survival of N3 stage SCLC patients may need to be further explored. Moreover, the predictive effect of pLNs, LNR, and LODDS on survival in SCLC patients was evaluated. LODDS showed a better predictive effect than pLNs and LNR for OS and CSS in SCLC patients. It was reported that LODDS is a better predictor of survival than pLNs and LNR for lung cancer patients.^16^^,^^17^ However, both this study and previous studies have reported that LODDS alone is a poor predictor (AUC < 0.70) for survival. The combination of LODDS with other clinical indicators may improve the prediction of survival in SCLC patients. For example, Chao et al. developed a nomogram using LODDS, age, tumor size, sex, and radiation therapy for predicting SCLC survival with an AUC of 0.76.^17^ However, prediction of survival in SCLC patients may require more research due to the high degree of risk and mortality of SCLC.

The authors assessed the relationship between three lymph node status evaluation metrics, pLNs, LNR, and LODDS, and the survival of SCLC patients. LODDS was a better metric than pLNs and LNR for predicting survival in SCLC patients. However, some limitations should be noted. Firstly, some potential confounders such as the course and dose of radiotherapy/chemotherapy and possible changes in treatment during follow-up could not be obtained because of the SEER limitations. Secondly, smoking is a major risk factor for the development of LC, but the current study lacks smoking-related factors due to the absence of relevant records in the database. Thirdly, this study was a retrospective analysis, inherent selection bias was inevitable.

## Conclusions

The correlations of LNR, pLNs, and LODDS with survival in SCLC patients were investigated. In multivariable COX analysis, only high LODDS values were linked to poorer survival in SCLC patients, and this relationship was significant only in patients with LN stage N1 and N2, but not in patients with stage N3. In addition, LODDS 'was a better predictor than pLNs and LNR for survival in SCLC patients. LODDS may be a better predictor of survival compared to other LN assessments in SCLC patients.

## Notes

SCLC, Small Cell Lung Cancer; LN, Lymph Node; LNR, LN Ratio, pLNs, positive LN; LODDS, Logarithmic Odds of positive LN.

## Ethics approval and consent for participation

The current study was approved by the Institutional Ethics Committee of Xianyang Central Hospital (nº 2023-IRB-70).

## Publication consent

None.

## Data and material availability

Data could be obtained from the corresponding author if a reasonable request was made.

## Funding

None.

## CRediT authorship contribution statement

**Ting Gao:** Conceptualization, Investigation, Methodology, Data curation, Formal analysis, Resources, Software, Validation, Writing – original draft. **Yingxuan Chang:** Data curation, Formal analysis, Resources, Software, Investigation, Validation. **Hongmei Yue:** Conceptualization, Methodology, Project administration, Supervision, Validation, Writing – review & editing.

## Declaration of competing interest

The authors declare no conflicts of interest.

## Appendix Supplementary materials

Image, application 1
