# Supplementary material for: Association of log odds of positive lymph nodes with survival in patients with small cell lung cancer: Results from the SEER database
Source: Clinics (Sao Paulo). 2024 May 1;79:100369. doi: 10.1016/j.clinsp.2024.100369 (PMC11070598; doi:10.1016/j.clinsp.2024.100369)
Supplement: Supplementary file 1 [file mmc1.docx]

CLINICS-D-23-00778_Suplementary Material

**Supplementary Table 1** COX univariable regression analysis for screening confounders associated with OS and CSS in SCLC patients.

| **Variables** | **OS** |  | **CSS** |  |
| --- | --- | --- | --- | --- |
|  | **HR (95%CI)** | **P** | **HR (95%CI)** | **P** |
| Age |  |  |  |  |
| ≥65 | Ref |  | Ref |  |
| <65 | 0.77 (0.70-0.85) | <0.001 | 0.79 (0.71-0.87) | <0.001 |
| Sex |  |  |  |  |
| Female | Ref |  | Ref |  |
| Male | 1.23 (1.11-1.35) | <0.001 | 1.22 (1.11-1.35) | <0.001 |
| Race | | |  |  |
| American Indian/Alaska Native | Ref |  | Ref |  |
| Asian or Pacific Islander | 0.78 (0.43-1.42) | 0.416 | 0.76 (0.42-1.39) | 0.377 |
| Black | 0.86 (0.49-1.52) | 0.601 | 0.82 (0.46-1.45) | 0.493 |
| White | 0.81 (0.47-1.40) | 0.444 | 0.76 (0.44-1.32) | 0.336 |
| Marital status |  |  |  |  |
| Married | Ref |  | Ref |  |
| Single | 1.12 (1.00-1.24) | 0.044 | 1.09 (0.98-1.22) | 0.123 |
| Others | 1.13 (0.97-1.31) | 0.13 | 1.11 (0.95-1.30) | 0.204 |
| AJCC T |  |  |  |  |
| T1 | Ref |  | Ref |  |
| T2 | 1.13 (0.97-1.31) | 0.108 | 1.13 (0.97-1.32) | 0.122 |
| T3 | 1.47 (1.14-1.89) | 0.003 | 1.50 (1.16-1.95) | 0.002 |
| TX | 1.60 (1.41-1.83) | <0.001 | 1.65 (1.44-1.89) | <0.001 |
| AJCC N | | | |  |
| N1 | Ref |  | Ref |  |
| N2 | 1.36 (1.15-1.61) | <0.001 | 1.32 (1.11-1.58) | 0.002 |
| N3 | 2.02 (1.69-2.42) | <0.001 | 2.01 (1.67-2.42) | <0.001 |
| AJCC M | | | |  |
| M0 | Ref |  | Ref |  |
| M1 | 1.94 (1.76-2.15) | <0.001 | 2.05 (1.85-2.27) | <0.001 |
| MX | 1.53 (1.11-2.12) | 0.01 | 1.55 (1.10-2.17) | 0.012 |
| Grade |  |  |  |  |
| Grade I | Ref |  | Ref |  |
| Grade II | 1.07 (0.29-3.96) | 0.918 | 1.13 (0.31-4.17) | 0.855 |
| Grade III | 2.30 (0.74-7.22) | 0.152 | 2.23 (0.71-6.99) | 0.169 |
| Grade IV | 2.29 (0.74-7.16) | 0.153 | 2.17 (0.70-6.78) | 0.182 |
| Unknown/others | 2.88 (0.93-8.96) | 0.067 | 2.73 (0.88-8.48) | 0.082 |
| Tumor size | |  |  |  |
| <50 mm | Ref |  | Ref |  |
| >50 mm | 1.31 (1.17-1.47) | <0.001 | 1.31 (1.16-1.47) | <0.001 |
| Laterality |  |  |  |  |
| Left - origin of primary | Ref |  | Ref |  |
| Right - origin of primary | 1.33 (1.03-1.73) | 0.032 | 1.34 (1.03-1.76) | 0.032 |
| Others | 0.98 (0.88-1.08) | 0.676 | 0.98 (0.88-1.08) | 0.645 |
| Primary Site | |  |  |  |
| Main bronchus | Ref |  | Ref |  |
| Upper lobe, lung | 0.87 (0.74-1.02) | 0.085 | 0.86 (0.72-1.01) | 0.069 |
| Middle lobe, lung | 0.96 (0.73-1.25) | 0.763 | 0.97 (0.74-1.27) | 0.818 |
| Lower lobe, lung | 1.13 (0.93-1.36) | 0.212 | 1.11 (0.92-1.35) | 0.269 |
| Overlapping lesion of lung | 1.22 (0.82-1.83) | 0.33 | 1.09 (0.70-1.68) | 0.713 |
| Lung (NOS) | 1.19 (0.98-1.43) | 0.073 | 1.18 (0.97-1.43) | 0.094 |
| Surgery type | |  |  |  |
| Lobectomy | Ref |  | Ref |  |
| Local tumor destruction | 0.95 (0.13-6.82) | 0.962 | 1.01 (0.14-7.22) | 0.994 |
| No surgery | 2.04 (1.70-2.45) | <0.001 | 2.06 (1.70-2.49) | <0.001 |
| Pneumonectomy | 1.15 (0.63-2.07) | 0.652 | 1.13 (0.61-2.09) | 0.708 |
| Sublobectomy | 1.48 (1.10-1.99) | 0.01 | 1.53 (1.13-2.08) | 0.006 |
| Surgery (NOS) | 1.37 (0.67-2.80) | 0.39 | 1.31 (0.61-2.80) | 0.492 |
| Radiation |  |  |  |  |
| No | Ref |  | Ref |  |
| Yes | 0.52 (0.47-0.57) | <0.001 | 0.52 (0.47-0.57) | <0.001 |
| Chemotherapy | | | |  |
| No | Ref |  | Ref |  |
| Yes | 0.44 (0.39-0.50) | <0.001 | 0.46 (0.41-0.52) | <0.001 |

OS, Overall Survival; CSS, Cancer-Specific Survival; SCLC, Small Cell Lung Cancer; AJCC, American Joint Committee on Cancer; TX, T-staging is not judgmental; MX, M-staging is not judgmental; NOS, Not Otherwise Specified; Ref, Reference; HR, Hazard Ratio; CI, Confidence Interval.
